# Vitamin D status among adults in Germany – results from the German Health Interview and Examination Survey for Adults (DEGS1)

**DOI:** 10.1186/s12889-015-2016-7

**Published:** 2015-07-11

**Authors:** Martina Rabenberg, Christa Scheidt-Nave, Markus A. Busch, Nina Rieckmann, Birte Hintzpeter, Gert B.M. Mensink

**Affiliations:** Department of Epidemiology and Health Monitoring, Robert Koch Institute, Berlin, Germany; Berlin School of Public Health, Charité – Universitätsmedizin, Berlin, Germany; University Medical Center Hamburg-Eppendorf, Hamburg, Germany

**Keywords:** Vitamin D, Serum 25(OH)D, Determinants, Season, Northern latitude, Population survey, Germany

## Abstract

**Background:**

In 1998, more than half of the adult population in Germany had serum 25-hydroxy-vitamin-D [25(OH)D] levels below the common threshold of 50 nmol/l. Since then, there has been a lot of attention for vitamin D in the scientific community, the media and the general population and serum 25(OH)D levels may have increased as a consequence. With data from the ‘German Health Interview and Examination Survey for Adults’ (DEGS1) the current situation of vitamin D status can be analysed.

**Methods:**

DEGS1, a national health survey among adults in Germany conducted by the Robert Koch Institute between 2008 and 2011, included 6,995 persons with available serum 25(OH)D levels. We calculated the proportion of participants with serum 25(OH)D levels <30 nmol/l, 30- < 50 nmol/l and > =50 nmol/l overall and according to age, season and latitude of residence as well as percentiles of serum 25(OH)D according to month of examination. Determinants of vitamin D status were analysed with multiple linear regression models.

**Results:**

Mean serum 25(OH)D level was 45.6 nmol/l with no significant sex differences (p = 0.47). 61.6 % of the participants had serum 25(OH)D levels <50 nmol/l, 30.2 % had levels <30 nmol/l. During summer, half of the participants had levels > =50 nmol/l, during winter time, 25 % of the participants had levels <30 nmol/l. A significant latitudinal gradient was observed in autumn for men and in winter for women.

In multiple linear regression analyses, examination during winter time, residing in northern latitude, non-use of vitamin D supplements, low sport activity, high Body Mass Index (BMI) and high media consumption were independently and significantly associated with lower serum 25(OH)D levels in both sexes. In addition, among women, significant associations with lower 25(OH)D levels were observed for older age and lower socio-economic status, among men, for low vitamin D intake and more residential traffic.

**Conclusions:**

Serum 25(OH)D levels below the threshold of 50 nmol/l are still common among adults in Germany, especially during winter and spring and in higher latitudes. Potentially modifiable factors of poorer vitamin D status are higher BMI, lack of sport activity and high media use.

## Background

Vitamin D plays an important role in the maintenance of calcium and phosphorus metabolism and therefore in bone health [24]. Moreover, in recent years, many epidemiological studies have reported associations between low levels of vitamin D and various non-skeletal chronic diseases, including diabetes mellitus, cardiovascular diseases, autoimmune diseases as well as lung, breast and colon cancer [25, 26, 34, 52].

The vitamin D status depends mainly on the production of vitamin D3 in the skin under the influence of UVB radiation (wavelengths 290–315 nm) and to a lesser degree on the intake of vitamin D2 and D3 through diet and dietary supplements [4, 28]. As sunlight exposure is the most important determinant for synthesis [26], endogenous vitamin D production is limited in northern latitudes during autumn and winter because of inadequate UVB radiation [13, 65]. This is also the case in Germany which is geographically located between latitudes 47° and 55°. In addition, the usual vitamin D intake from food in Germany does not meet the recommendations of the German Nutrition Society of 20 μg per day [16, 23, 57]. Based on data from the previous national health survey for adults (German National Health Interview and Examination Survey, GNHIES98) more than half of the adult population in Germany has serum 25-hydroxy-vitamin-D [25(OH)D] levels below the common threshold of 50 nmol/l [23].

In the past decade, public awareness of potential health effects of vitamin D deficiency has increased due to intense scientific debate and media coverage. As a consequence, 25(OH)D levels may have increased in the German population due to behaviour change in the general population and increased testing and prescribing of supplements among physicians. Support for this hypothesis is indicated by the doubling of unit sales of prescription and over-the-counter vitamin D preparations between 2008 and 2013 [29]. Due to potential changes of serum 25(OH) levels in the population, monitoring of the vitamin D status and its determining factors is necessary to provide a regular basis to form, implement and evaluate regulations as well as public health strategies on German and international level.

Against this background, the aim of the present study, the ‘German Health Interview and Examination Survey for Adults’ (DEGS1), is to give an overview of the current situation of vitamin D status and important determinants in the resident population 18–79 years of age in Germany. This comprises the analysis of serum 25(OH)D levels overall and according to sex, age, season and latitude of residence as well as the calculation of serum 25(OH)D percentile values according to month of examination. Finally, potential determinants of vitamin D status, including socio-demographic variables and indicators of health and health-related behaviours are considered by using multiple linear regression models.

## Methods

### Study design and subjects

**Fig. 1:**
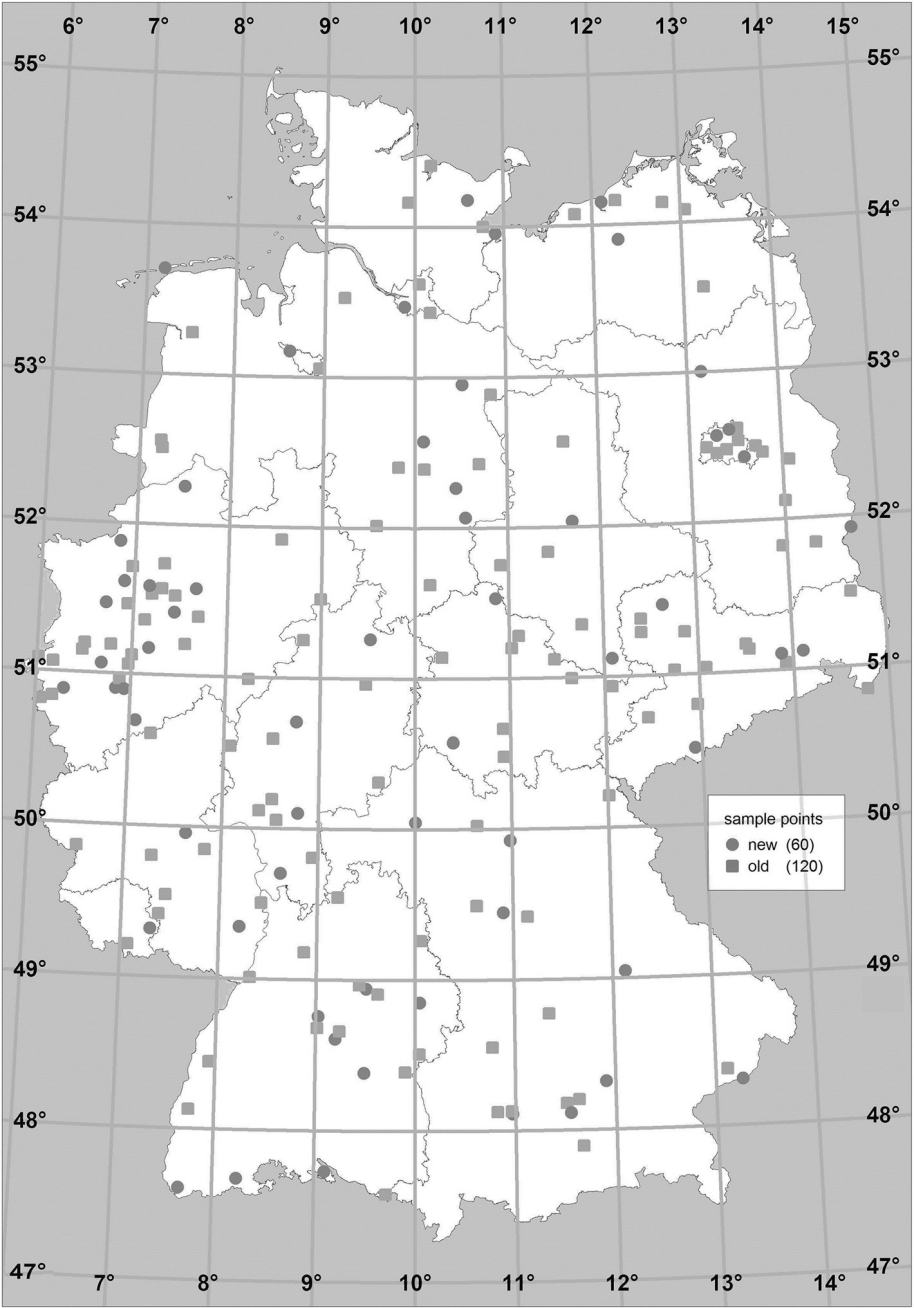
Map of Germany with DEGS1 sample points and geographic coordinates

From November 2008 to December 2011, DEGS1 was conducted by the Robert Koch Institute (RKI). DEGS1 is a comprehensive nationwide health survey among a representative population-based sample of adults 18–79 years old in Germany [58]. It includes both newly recruited participants as well as persons who already participated in the ‘German National Health Interview and Examination Survey 1998’ (GNHIES98) [3]. Overall, 8,151 adults (4,283 women; 3,868 men) with permanent residence in Germany participated in DEGS1. The sample included 3,959 persons from GNHIES98 (response rate 62 %) and 4,192 persons who were newly recruited by two-stage stratified random sampling (response rate 42 %). The sampling method and the study protocol were previously described in detail [36, 58]. In brief, at the first stage of sampling, a total of 180 sample points was randomly selected and stratified to reflect the federal state and community size including 120 original sample points of GNHIES98 and 60 new sample points (Fig. 1). At the second stage, participants were randomly selected from local population registries stratified by 5 year age groups.

Because the present analyses are restricted to participants with available serum 25(OH)D levels, the final study population consisted of 6,995 persons, 3,635 women and 3,360 men.

DEGS1 was developed in line with the principles of the Declaration of Helsinki and the German Federal Data Protection Act. The study protocol was approved by the Federal and State Commissioners for Data Protection and by the ethics committee of the Charité-Universitätsmedizin Berlin (No. EA2/047/08). All participants provided written informed consent prior to the study procedures.

### Data collection and operationalization of variables

In DEGS1, survey instruments comprised self-administered questionnaires, standardised computer-assisted interviews, physical examinations and tests, including blood sampling [58].

For analysis of serum 25(OH)D levels, venous blood samples were drawn with Vacutainer EDTA and gel tubes and immediately centrifuged and separated. Serum specimens were aliquoted and stored at −40 °C within one hour. For storage and detailed analysis, extra serum and whole blood samples were brought to the central epidemiology laboratory unit at the Robert Koch Institute, Berlin. Serum 25(OH)D was measured by a Liaison chemiluminescence immunoassay (DiaSorin Inc., Stillwater, MN, USA). The device ‘Liaison Analyzer’ had to be replaced in March 2011 due to a technical problem, without any other change in the analytical system. Interassay coefficients of variation measured in DEGS1 before March 2011 were 10.5 % for the lower part of the measuring range (37.2 nmol/l) and 10.3 % for the upper part of the measuring range (133.0 nmol/l). Following changes in test conditions, the interassay coefficients of variation improved slightly.

The lower detection level of the assay was 10 nmol/l. For analysis, measurement results >0 and <10 nmol/l (*n* = 111) were set to 9 nmol/l. In DEGS1, serum 25(OH)D levels were categorized as <30 nmol/l, 30- < 50 nmol/l and > =50 nmol/l based on currently available evidence summarized by the Institute of Medicine in 2011 [30]. Additionally, for some analyses, the categories 50- < 75 nmol/l and > =75 nmol/l were considered.

Month of examination was used to define season as spring (March to May), summer (June to August), autumn (September to November) and winter (December to February).

Latitudes were derived from participants’ region of residence and divided into three categories: 47°-49°, 50°-51° and 52°-54° (Fig. 1). Although Germany has a small area at latitude 55°, it was not involved because there was no sample point in this region.

To estimate vitamin D intake by food, a vitamin D intake index was constructed. Food intake was assessed with a validated semi-quantitative, self-administered food frequency questionnaire (FFQ) [19], which considered the consumption frequencies and portion sizes of 53 food groups within a recall period of 4 weeks. For the index, the following food groups were taken into account: fish, eggs, milk, cheese, cream cheese, curd cheese, yoghurt, soured milk, butter, margarine, and ice-cream. The index was computed by multiplying food frequencies with portion quantities and nutrient contents of the comprised food groups. As the FFQ provides information on particularly broad food groups for which calculation of average vitamin D contents is difficult, we used detailed information on particular food consumption from the German Nutrition Survey 1998 (GeNuS1998) [46] and linked it with the corresponding vitamin D contents reported in the ‘German Nutrient Content Database’ [45]. Accordingly, we computed weighted average nutrient contents for the included food groups. Based on tertiles of the distribution for the index, vitamin D intake was classified as higher (> = 2.81 μg/day), intermediate (> = 1.65 and <2.81 μg/day) and low (<1.65 μg/day).

Detailed information on medication use (prescription or over-the-counter) within the past 7 days was collected based on standardised methods [39]. These included a computer-assisted personal medication interview by specifically trained staff and barcode scanning of original drug packages brought to the study centre for this purpose. Scanned barcodes were automatically linked to the WHO Anatomical Therapeutic Chemical Classification System (ATC) for documentation of various information *e.g.,* preparation name, indication group, or standard package size [39]. For the present analysis, we used information on all drugs likely to affect vitamin D metabolism, including vitamin D supplements, oral contraceptives and postmenopausal hormone therapy. Moreover, any drugs known to activate the pregnane-X-receptor [18] such as anticonvulsants and glucocorticoids were taken into account.

Further, we considered chronic diseases known to affect vitamin D metabolism. Medical history was obtained by study physicians based on personal computer-assisted interviews. Specifically, we took into account a lifetime history of physician diagnosed renal insufficiency, cirrhosis of the liver, inflammatory bowel disease, cardiovascular diseases (heart attack, coronary heart disease, stroke, and heart failure), epilepsy, and diabetes mellitus as well as a history of gastroduodenal ulcer within the past 12 months.

Standardised measurements of body weight and body height were obtained with participants wearing underwear and no shoes. Body Mass Index (BMI) was calculated as body weight in kg divided by squared body height in metres. BMI was then classified as underweight (<18.5 kg/m^2^), normal weight (18.5– < 25 kg/m^2^), overweight (25- < 30 kg/m^2^), and obesity (> = 30 kg/m^2^) according to the categories of the World Health Organization [71].

Sport activities were assessed by asking ‘How often do you participate in sports during one week?’, with the possible answers: ‘I don’t do any sports’, ‘less than 1 h’, ‘regularly 1 to 2 h’, ‘regularly 2 to 4 h’ or ‘regularly more than 4 h’ per week. For multiple linear regression analyses, this variable was grouped into ‘no activity’, ‘less than 2 h sports per week’ and ‘2 or more hours sports per week’.

Media use was assessed with the questions ‘How many hours of your leisure time do you spend using TV/DVD/video on average?’ and ‘How many hours of your leisure time do you spend using computer/Internet/computer games/game consoles on average?’. Answers were ‘never’, ‘up to 1 h’, ‘1 to 2 h’, ‘2 to 3 h’ and ‘3 or more hours’ per day. For multiple linear regression analyses, answers were grouped into ‘up to 1 h a day (including never)’, ‘1 to 3 h per day’ and ‘3 or more hours per day‘.

Information on traffic exposure at the home residence, as a proxy for air pollution, was collected by asking if the participants live near a ‘side road with very little traffic (service road with access only, track, traffic-calmed zone)’, ‘side road with a fair amount of traffic’, ‘side road with quite a lot of traffic’, ‘main or through road with heavy traffic’ or ‘through road with extremely heavy traffic’. For multiple linear regression analyses, these answers were categorized as follows: living near a road with ‘very little traffic’, ‘fair amount or quite a lot of traffic’, and ‘heavy or extremely heavy traffic’.

Using information on education, occupation, and household income of the participants, a three-stage index of socio-economic status (low, middle, high) was built [40].

### Statistical analyses

All analyses were performed with SPSS statistical software (version 20.0; SPSS, Chicago, IL, USA). A weighting factor was used which adjusts for different sampling probabilities within the design strata and corrects deviations in the sample from the German population structure (as on 31. December 2010), taking into account age, sex, region, nationality, community type and education. Furthermore, calculation of the weighting factor also considered re-participation probability of GNHIES98 participants based on a logistic regression model [36, 58].

Because the distribution of serum 25(OH)D levels was slightly skewed, a log-transformation was carried out. As distributions of transformed and untransformed levels were similar, the untransformed results are presented.

Means of serum 25(OH)D levels according to study group characteristics were calculated for men and women separately. Moreover, we calculated percentiles of serum 25(OH)D levels according to month of examination and the proportion of men and women with serum 25(OH)D levels <30 nmol/l, 30- < 50 nmol/l and > =50 nmol/l, overall and stratified by age group, season and latitude of residence.

Finally, multiple linear regression analysis stratified by sex was used to consider potential determinants of vitamin D status, including serum 25(OH)D levels as dependent variable and the following independent variables: age and BMI (as continuous variables), season, latitude, vitamin D intake index, vitamin D supplement use, oral contraceptive use, postmenopausal hormone therapy, socio-economic status, sport activity, media use, and residential traffic exposure (as categorical variables). Furthermore, in sensitivity analyses, we excluded stepwise participants who suffered from certain diseases or used specific drugs which can influence the vitamin D status. For all analyses, a p-value <0.05 based on two-sided tests was considered statistically significant.

## Results

Characteristics of the study population with means of serum 25(OH)D stratified by sex are shown in Table 1.

**Table 1:** Characteristics of the study population with serum 25(OH)D means in nmol/l and 95 % confidence intervals^a^

	Women (*n* = 3,635)				Men (*n* = 3,360)			
	n	%	Mean (nmol/l)	95 % CI	n	%	Mean (nmol/l)	95 % CI
Age group (in years)								
18-44	1277	35.1	49.6	(46.6-52.7)	1179	35.1	45.5	(42.2-48.8)
45-64	1452	39.9	44.1	(42.2-46.0)	1271	37.8	45.1	(42.6-47.6)
65-79	906	24.9	41.3	(39.1-43.5)	910	27.1	45.1	(41.5-47.6)
Season of examination								
Spring (March-May)	890	24.6	40.7	(37.6-43.9)	845	25.4	38.8	(35.1-42.4)
Summer (June-August)	770	18.3	58.7	(55.5-62.0)	694	18.0	61.9	(58.5-65.4)
Autumn (Sept.-Nov.)	1134	31.2	51.2	(48.2-54.2)	1073	32.4	51.5	(47.5-55.4)
Winter (Dec.-Feb.)	841	25.9	35.4	(33.2-37.5)	748	24.2	31.3	(29.1-33.5)
Latitude								
47°-49°	1058	31.6	48.9	(45.2-52.7)	1000	31.2	49.3	(44.8-53.8)
50°-51°	1658	44.5	44.1	(41.4-46.7)	1601	44.5	42.5	(39.1-45.9)
52°-54°	919	23.8	45.3	(40.5-50.1)	759	24.3	45.1	(39.7-50.5)
Vitamin D intake index								
Low intake	1322	39.8	45.4	(42.9-47.9)	941	30.3	42.3	(39.4-45.2)
Intermediate intake	1205	32.9	45.7	(43.1-48.3)	1067	31.3	46.5	(43.4-49.6)
Higher intake	1019	27.3	46.4	(43.8-49.0)	1242	38.4	46.7	(44.0-49.5)
Use of vitamin D supplements								
Yes	221	5.9	55.9	(51.7-60.2)	45	1.0	60.3	(52.5-68.1)
No	3414	94.1	45.3	(43.2-47.4)	3315	99.0	45.1	(42.6-47.7)
Oral contraceptive use								
Yes	480	15.3	54.5	(49.9-59.1)	/	/	/	/
No	3155	84.7	44.3	(42.4-46.3)	/	/	/	/
Use of postmenopausal hormone therapy								
Yes	173	4.0	47.4	(43.3-51.6)	/	/	/	/
No	3462	96.0	45.8	(43.7-47.9)	/	/	/	/
Body Mass Index (kg/m^2^)								
<18.5	67	2.4	46.1	(38.7-53.6)	24	0.7	36.0	(26.9-45.1)
> = 18.5- < 25	1561	44.8	51.2	(48.4-54.0)	1011	32.1	47.5	(44.5-50.6)
> = 25- < 30	1111	29.2	44.1	(41.8-46.3)	1510	43.9	46.2	(43.5-48.9)
> = 30	867	23.6	37.6	(35.6-39.6)	796	23.3	40.2	(37.3-43.1)
Sport activity								
No sport activity	1119	33.3	41.4	(38.8-44.0)	1066	32.8	38.8	(36.5-41.2)
<2 h a week	1645	44.8	47.0	(44.7-49.2)	1268	38.1	46.6	(43.6-49.7)
2- < 4 h a week	545	14.9	52.3	(49.1-55.4)	518	15.6	51.1	(47.5-54.7)
> = 4 h a week	240	7.1	52.8	(47.5-58.1)	405	13.4	53.1	(49.6-56.7)
Media consumption per day								
TV, DVD, video								
up to 1 h	556	16.8	50.4	(46.7-54.0)	633	20.2	49.4	(45.6-53.2)
1- < 2 h	1223	34.9	47.9	(45.4-50.4)	1145	36.0	47.4	(44.4-50.4)
2- < 3 h	1224	33.5	44.7	(42.0-47.3)	1050	31.0	43.7	(41.0-46.5)
> = 3 h	547	14.8	40.0	(37.1-42.8)	427	12.7	39.5	(36.5-42.4)
Computer, Internet, computer games, game consoles								
up to 1 h	2571	74.9	46.6	(44.4-48.8)	1995	60.6	47.8	(45.2-50.4)
1- < 2 h	460	15.0	48.1	(44.5-51.7)	564	18.8	45.2	(41.3-49.1)
2- < 3 h	136	4.6	47.5	(40.9-54.1)	274	10.1	43.9	(39.8-48.0)
> = 3 h	159	5.5	44.2	(40.2-48.1)	265	10.5	40.3	(35.7-44.9)
Socio-economic status								
Low	587	20.0	39.5	(36.6-42.4)	516	19.0	40.7	(37.3-44.0)
Middle	2261	62.1	46.9	(44.5-49.2)	1924	58.7	46.0	(43.4-48.7)
High	765	17.9	50.3	(47.3-53.4)	897	22.3	47.7	(44.5-50.9)
Residential traffic								
Living near a road with…								
Very little traffic	1414	39.9	47.1	(44.6-49.5)	1354	40.7	47.7	(44.6-50.8)
Fair amount of traffic	988	27.1	46.9	(44.2-49.6)	935	28.8	45.3	(42.5-48.0)
Quite a lot of traffic	357	10.1	46.2	(42.4-50.1)	308	10.1	44.4	(40.0-48.7)
Heavy traffic	599	17.3	44.3	(41.1-47.6)	481	15.1	43.9	(40.7-47.1)
Extremely heavy traffic	177	5.6	44.3	(37.6-51.0)	173	5.4	40.3	(36.5-44.2)

*25(OH)D* 25-hydroxy-vitamin D, *CI* confidence interval

^a^ Results are weighted, except the number of cases

Overall, 48.0 % of the study population were men and 52.0 % were women. There were only few persons with vitamin D supplement intake during the last seven days (women: 5.9 %; men: 1.0 %) and vitamin D intake was higher among men than women. In both sexes, approximately one-quarter of adults 18–79 years were obese, and one-third did not engage in any sport activity. Furthermore, the majority of the study population lived in central Germany (latitude 50°-51°) and near roads with very little traffic.

**Fig. 2:**
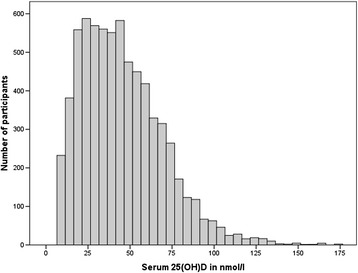
Distribution of serum 25(OH)D levels measured in DEGS1

The distribution of serum 25(OH)D levels measured in DEGS1 is illustrated in Fig. 2.

Levels ranged from 9 nmol/l to 347 nmol/l. The mean serum 25(OH)D concentration was 45.6 nmol/l (43.5–47.7) with no significant differences between men and women (45.3 nmol/l *vs.* 45.9 nmol/l; p-value = 0.47). Serum 25(OH)D categories by sex and age groups are shown in Table 2. The proportion of serum 25(OH)D levels <50 nmol/l increased from 55.9 % in women aged 18 to 44 years to 69.9 % in women aged 65 and older. In men, there were no pronounced age-differences: the proportion only slightly increased from 61.6 % in the youngest to 62.6 % in the oldest age group.

**Table 2:** Prevalence of serum 25(OH)D categories by sex and age group with 95 % confidence intervals

	<30 nmol/l	30- < 50 nmol/l	50- < 75 nmol/l	> = 75 nmol/l
Women (total)	29.7 % (26.5-33.1)	31.8 % (29.7-33.9)	26.6 % (24.1-29.2)	11.9 % (10.2-14.0)
18-44 years	28.9 % (24.8-33.4)	27.0 % (24.0-30.1)	26.6 % (23.2-30.3)	17.5 % (14.5-21.0)
45-64 years	28.8 % (25.1-32.7)	34.5 % (31.5-37.6)	27.9 % (24.8-31.3)	8.9 % (7.2-11.0)
65-79 years	32.9 % (28.3-37.9)	36.9 % (32.8-41.3)	24.4 % (20.5-28.8)	5.7 % (4.0-8.1)
Men (total)	30.8 % (26.8-35.2)	30.9 % (28.4-33.6)	26.6 % (23.8-29.7)	11.6 % (9.4-14.2)
18-44 years	32.8 % (27.9-38.2)	28.7 % (25.4-32.4)	24.2 % (21.1-27.7)	14.2 % (11.2-17.8)
45-64 years	30.4 % (25.9-35.3)	31.1 % (27.8-34.6)	28.3 % (24.6-32.4)	10.2 % (7.9-13.0)
65-79 years	26.6 % (21.8-32.2)	36.0 % (31.5-40.7)	29.1 % (24.7-34.1)	8.2 % (6.1-11.0)
Total	30.2 % (26.9-33.8)	31.3 % (29.4-33.3)	26.6 % (24.3-29.1)	11.8 % (10.0-13.9)

Table 3 gives an overview of percentile values according to month of examination stratified by sex. The data show a clear variation in 25(OH)D levels according to month of examination. Between June and September, half of the women had levels > =50 nmol/l, half of the men between June and October. On the other hand, between November and April, 25 % of the men had levels <30 nmol/l, 25 % of the women between November and May.

**Table 3:** Percentiles of serum 25(OH)D in nmol/l according to month of examination

Month of examination	5th		25th		Median		75th		95th	
	Women	Men	Women	Men	Women	Men	Women	Men	Women	Men
January	9.0	9.0	19.0	20.0	33.0	31.0	44.0	44.0	74.4	64.5
February	10.0	9.0	20.0	15.1	27.0	23.7	43.0	36.0	71.0	67.0
March	11.0	11.0	20.0	18.0	30.0	27.0	42.0	38.5	73.0	67.0
April	13.6	17.0	25.0	25.0	38.0	36.0	54.0	50.5	84.0	75.0
May	16.0	20.6	26.0	32.5	44.0	43.3	58.4	58.0	92.8	89.5
June	23.0	22.0	42.0	42.0	54.0	57.0	68.0	69.0	99.6	97.2
July	25.0	36.4	44.0	55.0	59.7	65.0	78.0	83.2	112.9	106.0
August	24.0	25.0	41.0	43.0	55.9	57.0	73.0	75.0	106.0	96.2
September	19.0	28.0	39.2	44.0	57.0	58.1	75.0	78.0	112.1	112.0
October	17.0	17.0	32.0	36.0	49.0	51.0	62.0	66.0	91.0	87.0
November	11.0	15.0	27.5	25.0	40.0	39.0	53.0	50.3	86.0	70.1
December	12.0	13.0	23.0	21.0	35.0	29.0	50.3	45.0	77.0	69.0

**Fig. 3:**
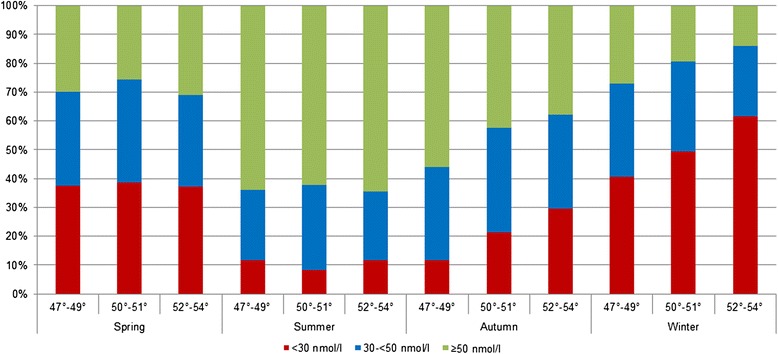
Proportion of 25(OH)D categories by season and latitudes in women

**Fig. 4:**
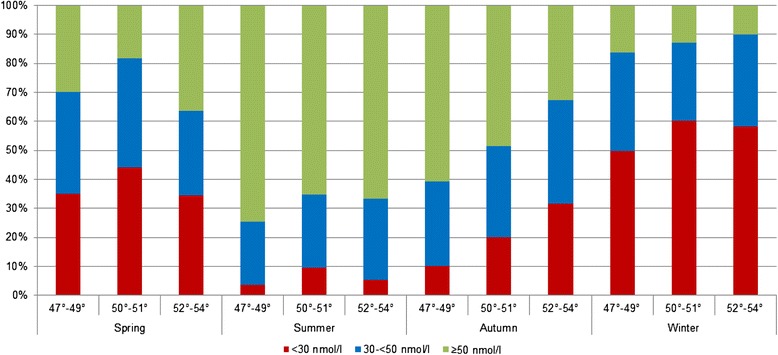
Proportion of 25(OH)D categories by season and latitudes in men

Figures 3 and 4 demonstrate the proportions of serum 25(OH)D categories by season and latitude. A clear south–north gradient with significant differences between the latitudes was only observed in autumn for men and in winter for women (data not shown).

Among men, significant differences were seen between latitudes 47°-49° and 50°-51° in every season, among women only in autumn and winter. Between latitudes 47°-49° and 52°-53°, significant differences were observed in autumn for both sexes and, additionally, in winter for women. Especially in spring and summer, persons residing in latitudes 50°-51°showed lower serum levels than their counterparts residing in higher latitudes. However, these differences were only statistically significant in men during springtime.

In multiple linear regression analyses, the following variables were significantly associated with higher serum 25(OH)D levels in both, men and women: summer, spring and autumn (reference category (ref.) winter), residing in latitudes 47°-49° (ref. 52°-54°), vitamin D supplement use, and sport activity up to and ≥ 2 h a week (ref. no activity) (Tables 4 and 5). In both sexes, higher BMI and use of computer, Internet, computer games and game consoles of > = 3 h per day (ref. up to one hour per day) were significantly associated with lower serum 25(OH)D levels (Tables 4 and 5).

**Table 4:** Results of a multiple linear regression analysis on determinants of serum 25(OH)D in women (*n* = 3,004)

Variable	B	SE	*p*-value
Intercept	51.535	2.946	0.000
Age (per year)	−0.132	0.032	0.000
Season (ref. winter)			
Spring	5.860	1.182	0.000
Summer	24.515	1.269	0.000
Autumn	16.847	1.118	0.000
Latitude (ref. 52°-54°)			
47°-49°	4.286	1.137	0.000
50°-51°	−0.005	1.050	0.996
Vitamin D intake index (ref. low intake)			
Intermediate intake	−0.378	0.979	0.700
Higher intake	2.013	1.041	0.053
Vitamin D supplement use	13.015	1.816	0.000
Oral contraceptive use	7.081	1.252	0.000
Use of postmenopausal hormone therapy	3.572	2.154	0.097
Body Mass Index (kg/m^2^)	−0.709	0.081	0.000
Socio-economic status (ref. low)			
Middle	4.467	1.116	0.000
High	4.575	1.405	0.001
Sport activity (ref. no activity)			
<2 h per week	2.676	0.971	0.006
> = 2 h per week	7.243	1.171	0.000
Media use (ref. up to 1 h per day)			
TV, DVD, video			
1- < 3 h	−0.773	1.134	0.495
> = 3 h	−2.294	1.564	0.142
Computer, Internet, computer games, game consoles			
1- < 3 h	−1.005	1.094	0.359
> = 3 h	−4.861	1.904	0.011
Residential traffic			
Living near a road with…			
(ref. very little traffic)			
Fair amount or quite a lot of traffic	−0.433	0.938	0.644
Heavy or extremely heavy traffic	−2.076	1.096	0.058
corr. r^2^ = 0.226			

*25(OH)D* 25-hydroxy-vitamin D, *ref*. reference

**Table 5:** Results of a multiple linear regression analysis on determinants of serum 25(OH)D in men (*n* = 3,021)

Variable	B	SE	*p*-value
Intercept	33.634	3.351	0.000
Age (per year)	0.044	0.029	0.125
Season (ref. winter)			
Spring	7.613	1.145	0.000
Summer	29.455	1.244	0.000
Autumn	19.454	1.081	0.000
Latitude (ref. 52°-54°)			
47°-49°	5.769	1.088	0.000
50°-51°	−0.496	1.004	0.621
Vitamin D intake index (ref. low intake)			
Intermediate intake	2.867	1.023	0.005
Higher intake	3.289	0.979	0.001
Vitamin D supplement use	10.891	4.767	0.022
Body Mass Index (kg/m^2^)	−0.337	0.095	0.000
Socio-economic status (ref. low)			
Middle	1.228	1.113	0.270
High	−0.505	1.331	0.704
Sport activity (ref. no activity)			
<2 h per week	6.773	0.974	0.000
> = 2 h per week	10.692	1.068	0.000
Media use (ref. up to 1 h per day)			
TV, DVD, video			
1- < 3 h	−1.729	1.014	0.088
> = 3 h	−5.634	1.472	0.000
Computer, Internet, computer games, game consoles			
1- < 3 h	−2.153	0.956	0.024
> = 3 h	−6.422	1.429	0.000
Residential traffic			
Living near a road with…			
(ref. very little traffic)			
Fair amount or quite a lot of traffic	−2.469	0.891	0.006
Heavy or extremely heavy traffic	−1.978	1.085	0.068
corr. r^2^ = 0.274			

*25(OH)D* 25-hydroxy-vitamin D, *ref*. reference

Among women, higher serum 25(OH)D levels were positively associated with use of oral contraceptives, and having an intermediate or high socio-economic status (compared to a low status) (Table 4). Moreover, higher age was significantly associated with lower serum levels of serum 25(OH)D.

Among men, dietary vitamin D intake was also significantly associated with higher serum 25(OH)D whereas use of TV, DVD and video of > = 3 h per day (ref. up to one hour per day), use of computer, Internet, computer games and game consoles of > = 1 h per day (ref. up to one hour per day) as well as living near a road with fair amount or quite a lot of traffic (ref. very little traffic) were associated with lower levels (Table 5).

These associations persisted in multiple linear regression analyses even after sensitivity analyses (data not shown).

## Discussion

In the German Health Interview and Examination Survey for Adults (DEGS1), the mean serum 25(OH)D level was 45.6 nmol/l with no significant sex differences (women: 45.9 nmol/l; men: 45.3 nmol/l). More than half of the population (61.6 %) had serum 25(OH)D levels <50 nmol/l, 30.2 % had levels <30 nmol/l, and 11.8 % had levels > =75 nmol/l.

In a subsample of 4,030 participants aged 18 to 79 years of the previous national health examination survey, the ‘German Health Interview and Examination Survey 1998’ (GNHIES98) [23], the proportion of persons having serum 25(OH)D levels <50 nmol/l was slightly lower with 56.8 % among men and 57.8 % among women. However, although the same methods of measurement were used to analyse serum 25(OH)D in both surveys, there were some methodological differences between GNHIES98 and DEGS1 which have to be considered while comparing the results. For example, in GNHIES98, the data collection period lasted one year compared to three years in DEGS1. Moreover, information on several potential determinants was assessed differently. Thus, only analyses including variables such as age, BMI, sport activity and postmenopausal hormone therapy can be compared.

However, other (smaller) German studies showed similar results regarding serum 25(OH)D levels. In the ‘DeViD-Trial’, a nationwide study that measured the vitamin D status of family practice patients, serum 25(OH)D levels below 50 nmol/l have been observed in 65 % of the 1,343 participants aged 20–99 years [56]. The study covered only four months in winter and spring which explains why serum 25(OH)D levels were lower than those measured in DEGS1 where blood samples were collected all year round. In the ‘Activity and Function in the Elderly in Ulm’ study (ActiFE Ulm), a population-based cohort study including 1,506 community-dwelling adults ≥65 years of age in southern Germany, mean 25(OH)D serum level was 50.1 nmol/l. After adjustment for sex, age and Body Mass Index, 78.8 % of the participants had 25(OH)D levels <50 nmol/l in March as compared to 16.1 % in August [37].

In addition to these findings, the results of DEGS1 are in line with outcomes of several other studies from European countries *e.g.,* the Netherlands [33], Denmark [64], France [11], Italy [31], Poland [49], and Switzerland [8]. Nevertheless, the comparison of vitamin D status of different studies is difficult. First, an evidence-based consensus on optimal levels of serum 25(OH)D has not yet been reached [27, 41], and, therefore, cut-offs used to evaluate vitamin D status (especially vitamin D deficiency and sufficiency) vary across different studies [5]. Second, although serum 25(OH)D is a valid and commonly used biomarker of vitamin D status, its measurement still lacks standardization [10, 61]. As a consequence, 25(OH)D measurements differ between studies due to differences in analytic methods, assays, and devices [9, 42, 54].

The vitamin D status depends mostly on the production of vitamin D in the skin under the influence of UVB radiation with wavelengths 290–315 nm. However, these wavelengths are only sufficiently available throughout the year in latitudes below 35°. In higher latitudes, the intensity and duration of adequate radiation decreases [69] and vitamin D status becomes dependent on season.

According to this, our results demonstrate seasonal as well as latitudinal differences in vitamin D status in Germany which is located between latitudes 47°-55°. Between June and September half of the women had levels > =50 nmol/l, half of the men between June and October. 25 % of the men had serum 25(OH)D levels <30 nmol/l from November to April, 25 % of the women from November to May. A somewhat delayed decrease of serum 25(OH)D levels in late autumn can be explained by the fat-soluble nature of vitamin D which makes it storable in the body’s fat tissue with a half-life of about four to six weeks [21].

Latitudinal differences were more pronounced among men than women, which may be due to unaccounted confounders, such as outdoor and indoor working, tanning habits, use of sun protection, and clothing style. Furthermore, latitudinal differences were evident during autumn and winter, but not in spring or summer. Remarkably, persons living in central Germany (lat. 50°-51°) had even lower levels than those living in northern Germany (lat. 52°-54°), in particular during spring and summer, although the differences were only statistically significant among men during spring time. Nevertheless, central Germany is more strongly urbanized which may be associated with a more indoor habitation. In addition, the WHO database on outdoor air pollution [70] shows that 7 out of 10 German cities with the highest annual mean concentration of particulate matter are located at latitudes 50°-51°. Air pollutants can absorb UVB radiation and therefore possibly reduce the cutaneous syntheses of vitamin D [43]. Apart from behavioural factors this may contribute to lower levels in central Germany. Accordingly, we used self-reported residential traffic exposure as a proxy for the amount of air pollution, but no consistent independent association with 25(OH)D was observed.

Age was significantly associated with lower levels of serum 25(OH)D among women, but not among men; a result which was also seen in former analyses of the GNHIES98 [23]. In general, older persons are at higher risk of lower levels *e.g.,* due to the reduced efficiency to produce vitamin D endogenously [26], the reduced capability to metabolize vitamin D in liver and kidneys [33], and less sunlight exposure [59]. Although the reasons for the age-specific sex differences are still unclear, an explanation might be a higher percentage of body fat in women [54], a greater avoidance of sunlight including increased sunscreen use and wearing covering clothing when going outside.

Dietary vitamin D intake, estimated by an vitamin D intake index, was significantly associated with serum 25(OH)D levels among men. This finding is not so obvious, since the dietary intake of vitamin D contributes only about 10–20 % to the vitamin D status [24]. In addition, data of GNHIES98 where individual nutrient intake was measured with a computerized modified dietary history instrument [47] showed that vitamin D intake among Germans is rather low (average intake per day among women: 2.4 μg; among men: 2.7 μg [23]) and does not meet the recommended daily intake of 20 μg [16]. Moreover, there was no significant association in multiple linear regression analyses between dietary intake and serum 25(OH)D in either sex [23].

However, the data from DEGS1 indicate that consumption of vitamin D-containing foods is associated with 25(OH)D serum levels, although the contribution may be low on average. In other studies [23, 57, 62, 66, 73], dietary vitamin D intake was found to be significantly associated with higher serum 25(OH)D levels among both sexes, whereas some studies found no association [35, 64]. A stronger relationship between vitamin D intake and vitamin D status was observed in countries where staple foods like milk, milk products, breakfast cereals, or orange juice are fortified with vitamin D, *e.g.,* in the US or Canada. In Germany, on the contrary, only few foods (*e.g.,* margarine) are voluntarily fortified with vitamin D.

Apart from this, the vitamin D intake depends on the use of dietary supplements. In DEGS1, only few persons (*n* = 266) used vitamin D-containing supplements. Nevertheless, the usage was significantly associated with higher vitamin D levels in women but not in men. In GNHIES98, such an association was not observed possibly due to a less specific assessment of vitamin D supplementation [23].

In DEGS1, other drugs found to be a determinant of serum 25(OH)D levels were oral contraceptives and postmenopausal hormone therapy medications. Consistent with other reports [15, 20, 22, 51, 62, 63], the present study shows that the intake of oral contraceptives in DEGS1 is significantly associated with higher levels of serum 25(OH)D. As a possible explanation for this, an oestrogen driven increase of 25(OH)D3-binding protein levels is discussed [55, 63]. Postmenopausal hormone therapy, however, showed no significant associations. National and international studies have provided conflicting results in this regard [23, 67]. Considering various other medications known to affect vitamin D metabolism in sensitivity analyses did not alter the results.

Higher BMI was significantly associated with lower serum 25(OH)D levels in men and women. In GNHIES98, such an association was not observed in multiple regression analyses [23], but similar results were reported in many other studies worldwide [7, 12, 14, 62, 64]. With data from the US ‘National Health and Nutrition Examination Survey’ (NHANES) Forrest *et al.* 2011 demonstrated that the risk of having serum 25(OH)D levels <50 nmol/l was about two times higher in obese than non-obese persons [14]. Daly *et al.* 2012 described that in obese men and women adjusted serum 25(OH)D levels were 8.3–9.5 nmol/l lower than in persons with normal weight [12]. Comparable results were found in an Italian study. Even after adjustment for High-Density Lipoprotein (HDL) cholesterol, Low-Density Lipoprotein (LDL) cholesterol, total cholesterol, triglycerides, and insulin-sensitivity BMI was significantly associated with serum 25(OH)D levels [48].

The reasons for the observed lower levels of serum 25(OH)D among person with higher BMI are still unclear. There is some evidence that vitamin D is accumulated in adipose tissue with decreased bioavailability and lower serum levels in persons with higher BMI [17, 72]. In addition, obese persons are possibly less exposed to sunlight, due to more indoor than outdoor activities and wearing skin-covering clothing when going outside [44, 72]. They may also have a less physically active lifestyle which is a determinant of obesity. Moreover, in epidemiological surveys, physical or sport activity is often used as a surrogate for time spent outdoors and, accordingly, for sun exposure. Hence, consistent with outcomes from other studies [6, 38, 53, 60, 66] our results showed that being physically active compared to being not active was significantly associated with higher serum 25(OH)D levels in both sexes. In GNHIES98, sport activity was also significantly associated with higher vitamin D levels in both men and women, although at a higher activity level of > = 2 h per week [23]. Since it was not possible to differentiate between indoor and outdoor sport activity, the variable gives probably only a rough estimate of time spent outdoors. However, results from some studies [2, 6] also suggest a physiological link between physical activity itself and 25(OH)D levels, as a positive association with 25(OH)D was observed for outdoor as well as indoor physical activities.

Moreover, a sedentary lifestyle with a high number of daylight hours spent indoors due to ‘nine-to-five’-jobs or indoor leisure time activities result in less sun exposure. Accordingly, in the present study use of computer, Internet, computer games and game consoles was significantly associated with lower vitamin D levels among both sexes. In addition, among men, significant associations were seen for use of TV, DVD and video.

We observed an independent and positive relationship between socio-economic status and serum 25(OH)D levels in women as compared to no association in men. Shirazi *et al.* 2013 who examined 727 Swedish women aged >40 years, only found a weak association with SES although there were slightly higher serum 25(OH)D levels in women working in manual than non-manual jobs [62]. Several other studies examined education or income. Most [14, 32, 50, 68] but not all of these [12] confirmed that persons with higher education or higher income have higher levels of serum 25(OH)D. Previous studies, however, did not differentiate between men and women. The results found in DEGS1 for SES may be confounded by outdoor work which is more seen among men than women. Men with lower SES may therefore have more similar results to their counterparts with higher SES.

Our study has some limitations. First of all, the results of multivariable analyses may be biased due to misclassification of sun exposure as the major determinant of vitamin D status. Although we accounted for season and latitude, we did not collect direct measures of individual sun exposure, such as the number of hours and time of day spent outdoors, sun protection habits (*e.g.,* avoidance of direct sun exposure, seeking shady places, use of sunscreen, wearing skin-covering clothing), travelling habits shortly before the survey visit or sunbed use [12, 22]. Furthermore, we could also not account for cutaneous pigmentation and cultural or religious dress codes such as veiling. In DEGS1, information on behavioural determinants was limited to the proxy variables sport activity and media use.

Second, food intake data were collected by a food frequency questionnaire which does not permit precise estimates of nutrient intake. Therefore, we linked the FFQ data on vitamin D- containing foods with weighted average nutrient contents which were estimated by using detailed data of the German Nutrition Survey 1998 (GeNuS1998) [46] and the ‘German Nutrient Content Database’ [45]. The vitamin D intake index is, however, still a relatively rough estimate of the dietary vitamin D intake.

Third, a short recall period of 7 days was used to assess current medication use. Although the recall bias might be reduced, the use of drugs which are taken intermittently, in particular oral contraceptives may be underestimated.

Finally, the present analysis is based on a cross-sectional study design and hence precludes any causal implications.

## Conclusions

In conclusion, we found that serum 25(OH)D levels below 50 nmol/l are (still) widespread in the German adult population aged 18–79 years, particularly in winter and spring and in higher latitudes. Thus, although there has been a lot of attention for vitamin D in the past decade, the average vitamin D status remained almost unchanged compared to the previous national health survey for adults in 1998.

Apart from that, in DEGS1 a significant south–north gradient was observed in autumn for men and in winter for women. With regard to potentially modifiable factors, the results of the present analysis confirm a higher probability of poorer vitamin D status for persons with higher BMI, lack of sport activity or higher media use.

Although a recent systematic review on the effect of 25(OH)D concentrations on non-skeletal health indicates that poor levels of vitamin D are rather a marker than a cause of ill health [1], the causal association with skeletal health is beyond controversy: low levels of serum 25(OH)D, particularly at levels <30 nmol/l, are associated with clinically adverse outcomes such as osteomalacia and osteoporosis [24]. Therefore, efforts should be made to maintain vitamin D status throughout the year by spending time outdoors regularly (without taking risks of sun burns and skin cancer), and paying attention to a healthy diet rich in vitamin D, especially during winter and spring.
